# Antimicrobial resistance and genetic diversity in ceftazidime non-susceptible bacterial pathogens from ready-to-eat street foods in three Taiwanese cities

**DOI:** 10.1038/s41598-017-15627-8

**Published:** 2017-11-14

**Authors:** Lin Lin, Sheng-Fan Wang, Tsung-Ying Yang, Wei-Chun Hung, Min-Yu Chan, Sung-Pin Tseng

**Affiliations:** 1 Department of Culinary Art, I-Shou University, Kaohsiung, Taiwan; 2 Department of Medical Laboratory Science and Biotechnology, Kaohsiung Medical University, Kaohsiung, Taiwan; 3 Department of Microbiology and Immunology, Kaohsiung Medical University, Kaohsiung, Taiwan; 4 Department of Marine Biotechnology and Resources, National Sun Yat-sen University, Kaohsiung, Taiwan

## Abstract

Bacterial contamination of ready-to-eat (RTE) street foods is a major concern worldwide. Dissemination of antibiotic resistant pathogens from food is an emerging public-health threat. To investigate the prevalence of antibiotic resistance genes and ceftazidime resistance-associated efflux pumps in foodborne pathogens, 270 RTE street foods samples were collected in three densely populated Taiwanese cities. Among 70 ceftazidime non-susceptible isolates, 21 *Stenotrophomonas maltophilia*, 12 *Pseudomonas* spp., 22 *Acinetobacter* spp., and 15 *Enterobacteriaceae* isolates were identified. Phylogenetic analyses revealed high levels of genetic diversity between all of the different strains. Multi-drug resistance was observed in 86.4% (19/22) of *Acinetobacter* spp., 100% (12/12) of *Pseudomonas* spp., 71.4% (15/21) of *S*. *maltophilia*, and 93.3% (14/15) of *Enterobacteriaceae*. Of 70 ceftazidime non-susceptible isolates, 13 contained ESBLs or plasmid-mediated *ampC* genes and 23 contained ceftazidime resistance-associated efflux pumps, with *Acinetobacter* spp. identified as predominant isolate (69.6%; 16/23). AdeIJK pump RNA expression in *Acinetobacter* isolates was 1.9- to 2-fold higher in active efflux strains. Nine clinically resistant genes were detected: *catIII* and *cmlA* (chloramphenicol); *aacC1*, *aacC2*, *aacC3*, and *aacC4* (gentamicin); *tet*(A), *tet*(C), and *tet*(D) (tetracycline). The scope and abundance of multidrug-resistant bacteria described in this report underscores the need for ongoing and/or expanded RTE monitoring and control measures.

## Introduction

Taiwan is one of many countries with a vibrant street food culture, with ready-to-eat (RTE) foods and snacks easily found at almost any time of day, and with night markets and food stands being major attractions for both domestic and international tourists^1–4^. According to one estimate, more than 150,000 types of food and drink items are sold on the streets and in night markets across the country^5^. Street food culture has always been associated with food poisoning and other gastrointestinal maladies that can also be transmitted in restaurants and various food service venues. According to data gathered by the Republic of China Food and Drug Administration, there was a significant increase in reported food poisoning cases between 1991–2000 (38,938) and 2000–2010 (43,404)^6^. Taiwanese citizens are clearly aware of this problem, and have organized efforts to get government agencies to deal with it.

RTE meats and salads have long been described as hidden vectors of microbial foodborne pathogens such as *Enterobacteriaceae* and *Listeria monocytogenes* ^7–9^. Many researchers have reported on the role of street foods as vectors of pathogenic bacterial transmission to humans. Whereas most studies focus on microbiological and hygienic quality, fewer efforts have been made to identify antibiotic resistance mechanisms and the dissemination of antibiotic resistance genes—important information in the fight against foodborne pathogens^10–13^. The literature contains several studies that assessed foodborne pathogens in RTE food in other countries. For example, among 154 foodborne *Staphylococcus aureus* isolates analyzed in Turkey, 39 (25.3%) were found to have multi-drug resistance^14^. In one study of vegetables collected in Switzerland, 78.3% (47/60) of multidrug-resistant extended-spectrum β-lactamase (ESBL)-producing *Enterobacteriaceae* isolates were identified^15^. ESBLs are enzymes that hydrolyze penicillin as well as aztreonam and first-, second-, and third-generation cephalosporins^16^. Although, third-generation cephalosporins are broad-spectrum antimicrobial agents against bacterial infection and useful for different type of clinical situations^17^, the problem of ESBL-producing bacteria has been identified in medical systems and communities worldwide^16,18^. A primary reason for this rapid spread is that ESBL-producing bacteria can be transmitted by contaminated food or water^9,19,20^.

In Taiwan, at least two research teams have focused on microbiological quality in RTE foods, but no efforts have been made to determine antibiotic resistance levels and associated resistance mechanisms in these foods^10,12^. Bacterial isolates containing ESBLs have been detected in hospital patients and livestock, though transmission mechanisms remain unidentified^21–24^. To determine the potential contribution of food to the dissemination of antibiotic resistant pathogens, we collected and analyzed 270 RTE street food samples in the densely populated cities of Kaohsiung, Taichung and Taipei, including spring rolls, cold noodles and fruit platters. One of our primary motivations was to determine the prevalence and mechanisms of antibiotic resistance, specifically the contribution of efflux pumps to ceftazidime resistance.

## Results

### Prevalence of ceftazidime non-susceptible bacterial isolates

**Figure 1:**
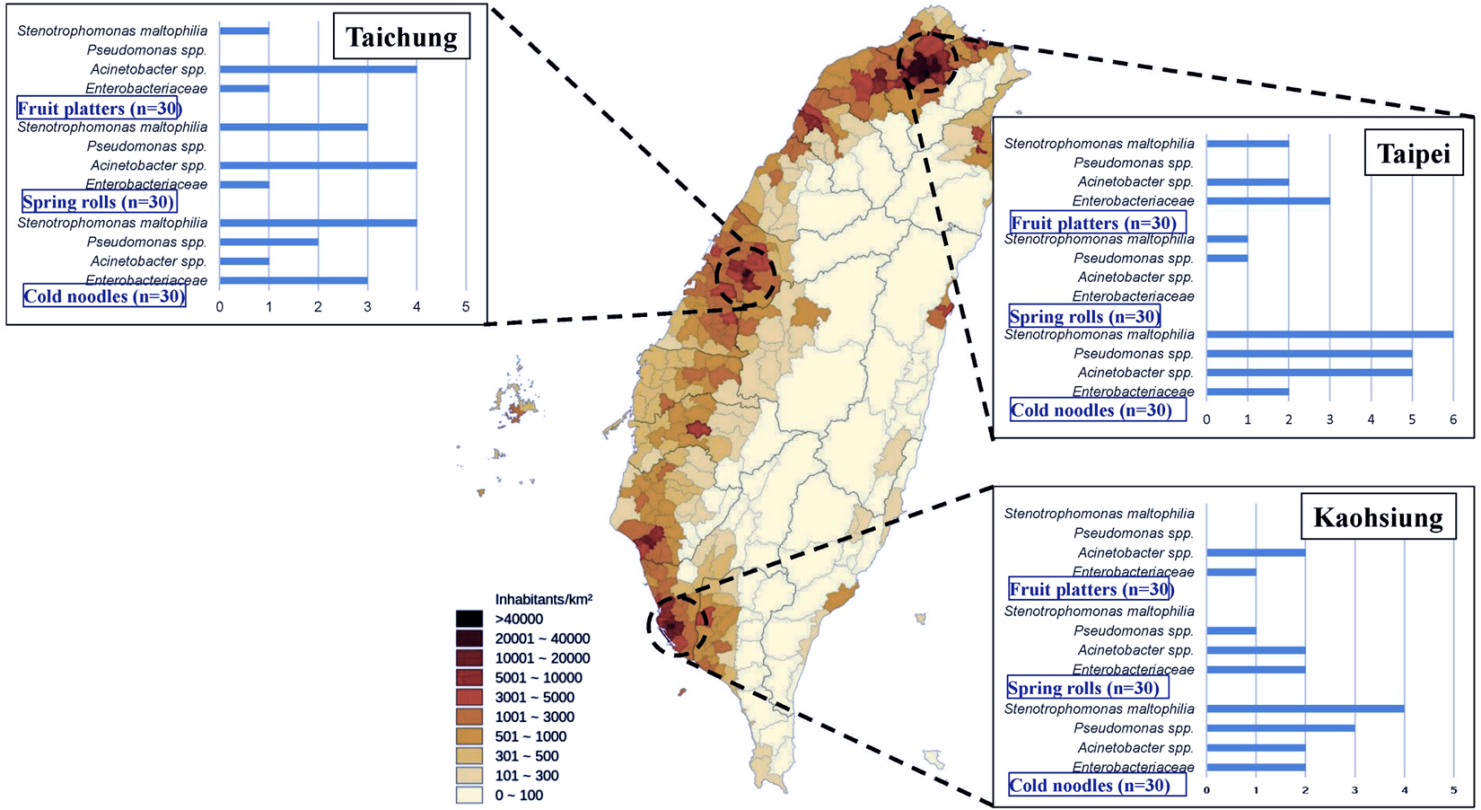
Map showing population densities of geographic locations throughout Taiwan, including the three cities where the RTE street food samples used in this study were collected: Kaohsiung, Taichung and Taipei. The map of population densities in Taiwan was modified from Kanguole (2013) via Wikimedia Commons (https://upload.wikimedia.org/wikipedia/commons/8/88/Population_density_of_Taiwan_by_district.svg). This file is licensed under the Creative Commons Attribution-Share Alike 3.0 Unported licensing agreement (https://creativecommons.org/licenses/by-sa/3.0/).

**Figure 2:**
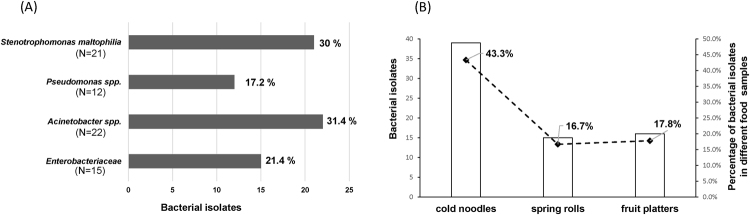
(**A**) Percentages of ceftazidime non-susceptibility among 70 bacterial isolates. (**B**) Distribution of ceftazidime non-susceptible bacterial isolates in the RTE samples tested for this project.

Of the 270 food samples (three RTE food types: spring rolls, cold noodles, and fruit platters) we tested, ceftazidime non-susceptible *Enterobacteriaceae*, *S*. *maltophilia*, *Pseudomonas* spp. and *Acinetobacter* spp. were selected by eosin-methylene blue (EMB) agar containing ceftazidime (8 μg/ml) and identified using 16S rRNA gene analysis (Fig. 1). Characterization of 70 bacterial isolates identified 21 instances of *S*. *maltophilia*, 12 *Pseudomonas* spp., 22 *Acinetobacter* spp. and 15 *Enterobacteriaceae* (Fig. 2A). The highest percentage of ceftazidime non-susceptible bacteria was isolated in cold noodle samples (43.3%; 39 of 90); the spring rolls and fruit platters we tested had similar levels of isolates (16.7% and 17.8%) (Fig. 2B). The lowest percentages were in spring rolls collected in Taipei (1 *Pseudomonas aeruginosa* and 1 *S*. *maltophilia*) and fruit platters collected in Kaohsiung (1 *Enterobacter cloacae* and 2 *Acinetobacter baumannii*) (Fig. 1).

### Antimicrobial susceptibility testing

**Figure 3:**
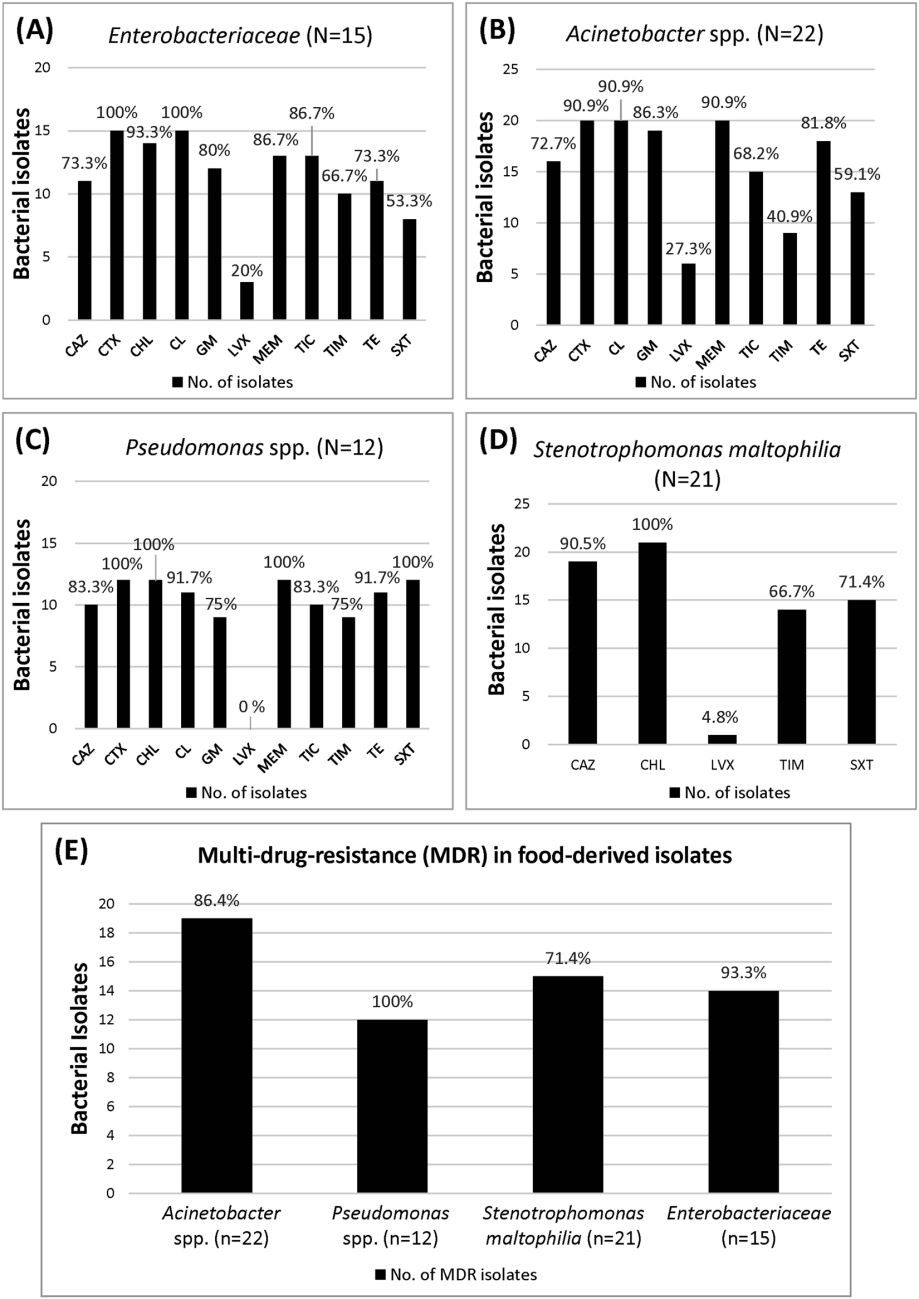
Percentages of antibiotic resistance in 70 bacterial isolates analyzed in this study. CAZ, ceftazidime; CTX, cefotaxime; CHL, chloramphenicol; CL, colistin; GM, gentamicin; LVX, levofloxacin; MEM, meropenem; TIC, ticarcillin; TIM, ticarcillin-clavulanic acid; TE, tetracycline; SXT, trimethoprim-sulfamethoxazole. Shown are bacterial isolate and resistance percentage data for (**A**) *Enterobacteriaceae* (N = 15), (**B**) *Acinetobacter* spp. (N = 22), (**C**) *Pseudomonas* spp. (N = 12) and (**D**) *S*. *maltophilia* (N = 21). Further details are shown in Supplementary Table S2.

As shown in Fig. 3, we observed high levels of antibiotic resistance (73.3–100%) among 15 *Enterobacteriaceae* isolates (Fig. 3A). The lowest level of resistance was for levofloxacin (20%) and the highest for trimethoprim-sulfamethoxazole (53.3%). We observed 27.3–90.9% antibiotic resistance in 22 *Acinetobacter* spp. isolates (Fig. 3B). Resistance levels for ticarcillin and ticarcillin-clavulanic acid were 68.2% and 40.9%, respectively. According to this finding, combinations of these antibiotics are capable of increased efficacy against ticarcillin-resistant *Acinetobacter* spp. Among the 12 *Pseudomonas* spp. isolates, high levels of antibiotic resistance (75–100%) were found in 9 (Fig. 3C); all 12 were susceptible to levofloxacin. Among the 21 *S*. *maltophilia* isolates, we noted high resistance levels (71.4–100%) against 3 antibiotics (Fig. 3D). The ticarcillin-clavulanic acid resistance level for *S*. *maltophilia* was 66.7%; only 1 isolate was found to be levofloxacin-resistant.

For this study, multidrug resistance was defined as resistance to a minimum of 3 antibiotics. Our data indicate the presence of multidrug resistance in 86.4% (19/22) of *Acinetobacter* spp., 100% (12/12) of *Pseudomonas* spp., 71.4% (15/21) of *S*. *maltophilia*, and 93.3% (14/15) of *Enterobacteriaceae* isolates (Fig. 3E). These high percentages indicate that RTE street foods in Taiwan may be a significant reservoir for antibiotic resistant pathogens.

### Investigation of ceftazidime non-susceptible bacterial isolates

Phylogenetic typing identified 20 pulsotypes in 22 *Acinetobacter* spp. isolates. Two isolates sharing the same pulsotype, TP36 and TP47, were identified in two fruit platter samples collected in Taipei (Supplementary Fig. S1). PFGE analysis of 12 *Pseudomonas* spp., 21 *S*. *maltophilia*, and 15 *Enterobacteriaceae* isolates indicate that they were non-clonal (Supplementary Figs S2–S4). The high levels of genetic diversity and multi-drug resistance identified among these bacterial isolates suggest that they developed antibiotic resistance mechanisms independently in the three cities where samples were collected.

### Ceftazidime resistance mechanism

Among the 70 ceftazidime non-susceptible isolates that were the focus of this study, 13 contained ESBLs or plasmid-mediated *ampC* genes (Table 1). *bla*_PER-1_ was found in 5 *Acinetobacter* spp. isolates and *bla*_CTX-M-9_ was detected in *E*. *coli* (n = 1), *S*. *maltophilia* (n = 1), and *A*. *baumannii* (n = 1). This suggests that *bla*_CTX-M-9_ is horizontally transferred among various bacterial species.

**Table 1:** ESBL and plasmid-mediated *ampC* genes associated with RTE food-derived isolates in Taiwan (n = 13).

Sample ID	Source	Location	Species	ESBL/*ampC*
**TC-23**	Cold noodles	Taichung	*E*. *coli*	*bla*_DHA_, *bla*_CTX-M-9_
**TC-90**	Spring rolls	Taichung	*Klebsiella pneumoniae*	*bla*_DHA_
**TP-163**	Fruit	Taipei	*S*. *maltophilia*	*bla*_CTX-M-9_
**KA-75**	Spring rolls	Kaohsiung	*Acinetobacter soli*	*bla*_PER-1_
**TC-167**	Fruit	Taichung	*Acinetobacter calcoaceticus*	*bla*_OXA-10_, *bla*_PER-1_
**TC-172**	Fruit	Taichung	*Acinetobacter radioresistens*	*bla*_SHV_, *bla*_PER-1_
**TC-175**	Fruit	Taichung	*Acinetobacter radioresistens*	*bla*_PER-1_
**TC-177**	Fruit	Taichung	*Acinetobacter radioresistens*	*bla*_PER-1_
**TC-98**	Spring rolls	Taichung	*Acinetobacter baumannii*	*bla*_CTX-M-9_
**TC-91**	Spring rolls	Taichung	*Acinetobacter calcoaceticus*	*bla*_DHA_
**TC-126**	Spring rolls	Taichung	*Acinetobacter baumannii*	*bla*_OXA-10_
**TP-179**	Cold noodles	Taipei	*Pseudomonas geniculata*	*bla*_CMY-2_
**TP-157**	Spring rolls	Taipei	*Pseudomonas putida*	*bla*_SHV_

**Figure 4:**
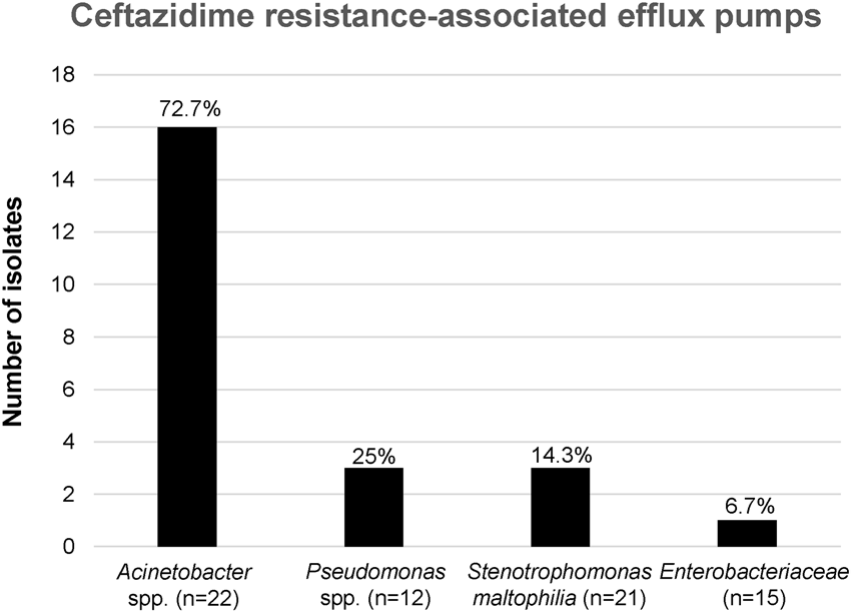
Efflux pump contributions to ceftazidime resistance in 70 non-susceptible bacterial isolates. Ceftazidime resistance was defined as a minimum four-fold decrease in MIC level in the presence of carbonyl cyanide-m-chlorophenyl hydrazine (CCCP). Number of strains are bacterial isolates with ceftazidime resistance-associated efflux pumps.

To clarify the contribution of efflux pumps to ceftazidime resistance, we used the efflux pump inhibitor CCCP to analyze the 70 ceftazidime non-susceptible isolates. Bacterial isolates containing ceftazidime resistance-associated efflux pumps were identified as any CCCP- inoculated strain exhibiting a minimum 4-fold decrease in minimum inhibitory concentration (MIC). Our data indicate that 72.7% (16/22) of the *Acinetobacter* spp. and 25% (3/12) of the *Pseudomonas* spp. isolates contained efflux pumps conferring resistance to ceftazidime (Fig. 4). A ceftazidime-induced effect on efflux activity was observed in only 3* S*. *maltophilia* isolates (14.3%) and 1 *Enterobacteriaceae* isolate (6.7%). These findings suggest a significant prevalence of ceftazidime resistance-associated efflux pumps in *Acinetobacter* spp.

### RNA expression of two efflux pumps in *Acinetobacter* spp

**Figure 5:**
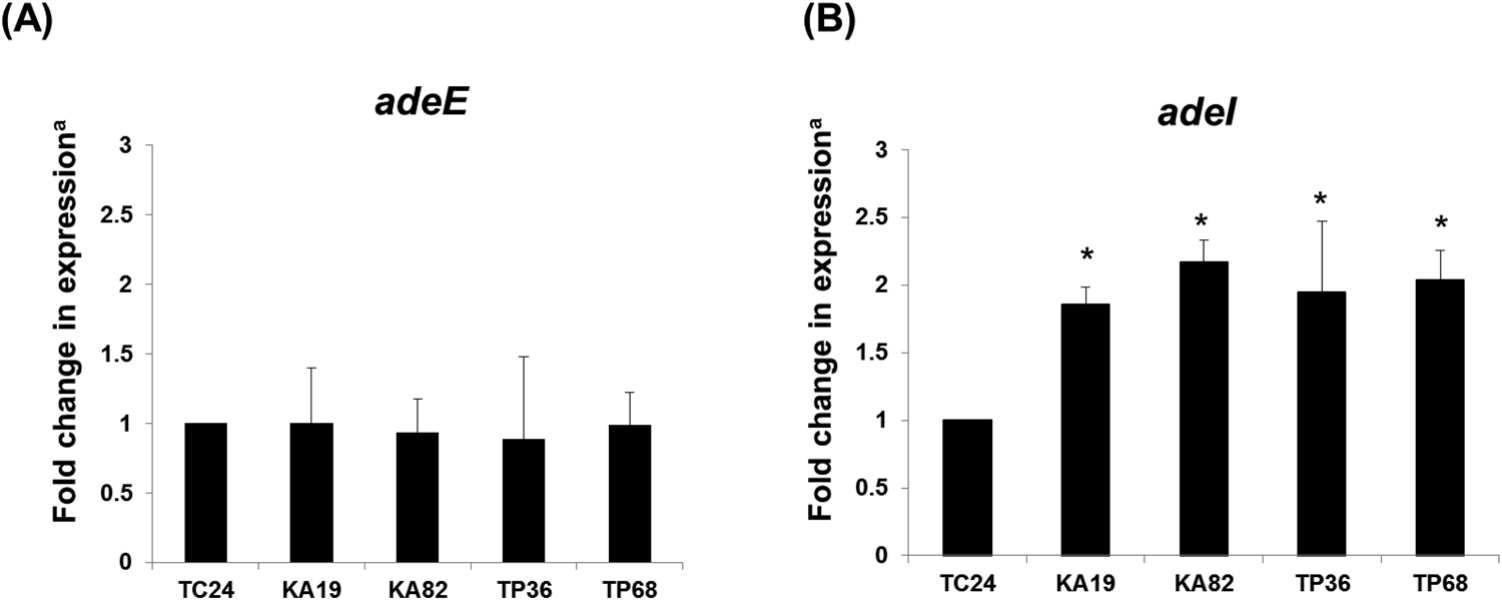
RNA expression levels of *adeDE* (*adeE*) and *adeIJK* (*adeI*) efflux pump systems in five representative isolates. ^a^Fold changes were determined by quantitative RT-PCR. TC24, a strain lacking efflux pump activity, served as a reference. *p < 0.05 (Mann-Whitney U tests).

Previous studies have determined a link between the active efflux pumps *adeDE* and *adeIJK* and resistance to third-generation cephalosporins in *Acinetobacter* spp.^25,26^ We analyzed expression of *adeDE* and *adeIJK* in four representative isolates exhibiting efflux pump activity (strains KA19, KA82, TP36, TP68) relative to a control strain that did not (strain TC24). Our data indicate that *adeE* expression was not significantly different from that of the control strain (Fig. 5A), while *adeI* expression was 1.9- to 2.2-fold (*p* < 0.05) higher than in the control strain (Fig. 5B). These results suggest that ceftazidime resistance in *Acinetobacter* spp. may be attributed to *adeI* expression levels.

### Antimicrobial resistance determinants

Among the 70 isolates, 4 contained chloramphenicol resistance genes, 7 gentamicin resistance genes, and 3 tetracycline resistance genes (Table 2). Among the 4 with chloramphenicol resistance genes, 1 *S*. *maltophilia* and 3 *Pseudomonas* spp. isolates encoded *catIII* and *cmlA* genes, respectively. Among the 7 containing gentamicin resistance genes, *aacC2* was identified in 3 (2 *Acinetobacter* spp. and 1 *Pseudomonas* spp.) and *aacC4* in 2 (1 *Acinetobacter* spp. and 1 *Enterobacteriaceae*). Two *Acinetobacter* spp. isolates also carried the gentamicin resistance genes *aacC1* and *aacC3*. Three tetracycline resistance genes (*tet*(A), *tet*(C) and *tet*(D)) were identified in three *Enterobacteriaceae* isolates.

**Table 2:** Antibiotic-resistance genes detected in the 70 bacterial strains isolated in this study.

Related Antibiotic	Species Genes	*Acinetobacter spp*. (n = 22) [n/N^b^ (%)]	*Pseudomonas spp*. (n = 12) [n/N^b^ (%)]	*S*. *maltophilia* (n = 21) [n/N^b^ (%)]	*Enterobacteriaceae* (n = 15) [n/N^b^ (%)]
chloramphenicol	*catI*	ND^a^	0/12 (0)	0/21 (0)	0/14 (0)
	*catII*	ND^a^	0/12 (0)	0/21 (0)	0/14 (0)
	*catIII*	ND^a^	0/12 (0)	1/21 (4.8)	0/14 (0)
	*cmlA*	ND^a^	3/12 (25)	0/21 (0)	0/14 (0)
gentamicin	*aacC1*	1/19 (5.3)	0/9 (0)	ND^a^	0/12 (0)
	*aacC2*	2/19 (10.5)	1/9 (11.1)	ND^a^	0/12 (0)
	*aacC3*	1/19 (5.3)	0/9 (0)	ND^a^	0/12 (0)
	*aacC4*	1/19 (5.3)	0/9 (0)	ND^a^	1/12 (8.3)
tetracycline	*tet*(A)	0/18 (0)	0/11 (0)	ND^a^	1/11 (9.1)
	*tet*(B)	0/18 (0)	0/11 (0)	ND^a^	0/11 (0)
	*tet*(C)	0/18 (0)	0/11 (0)	ND^a^	1/11 (9.1)
	*tet*(D)	0/18 (0)	0/11 (0)	ND^a^	1/11 (9.1)
	*tet*(E)	0/18 (0)	0/11 (0)	ND^a^	0/11 (0)
	*tet*(G)	0/18 (0)	0/11 (0)	ND^a^	0/11 (0)

^a^ND: non-detected. Criteria for chloramphenicol resistance in *Acinetobacter* spp. and both gentamicin and tetracycline resistance in *S*. *maltophilia* have not been defined by the Clinical Laboratory Standards Institute.

^b^n: numbers of antibiotic resistance gene-positive isolates. N: total numbers of antibiotic resistance isolates.

## Discussion

The literature contains studies of the microbiological quality of various RTE foods in countries around the world. Oliveira *et al*. tested 162 minimally processed leafy vegetable samples in Brazil and reported that 53.1% contained *E*. *coli*, 3.7% *Listeria* spp., and 1.2% *Salmonella* spp.^13^. In Argentina, the foodborne pathogens *Bacillus cereus*, *Clostridium perfringens*, *S*. *aureus* and *Salmonella* spp. were found in 101 RTE cooked food samples collected from the central kitchen facility of a school district^27^. The researchers focused on *B*. *cereus* (found in 63.4% of their samples) and reported bacterial counts of <4 log CFU/g. In a Portuguese study, Campos *et al*. described poor microbiological quality in a number of RTE food types and food products prepared by vendors working out of trailers: 100% tested positive for *Enterobacteriaceae* and coliforms, 20% for *E*. *coli* (4 hamburgers, 4 other trailer foods), and 20% for *L*. *monocytogenes* (2 hamburgers/2 hotdogs, 3 other trailer foods)^7^. In an Italian study of whole vegetables and RTE salads, *Salmonella* spp., *L*. *monocytogenes*, *E*. *coli* O157:H7, hepatitis A, and noroviruses were detected in 964 samples^28^. Of these, only 2 and 3 tested positive for *Salmonella* spp. and *L*. *monocytogenes*, respectively.

In one of two studies conducted in Taiwan, Fang *et al*. reported the following levels of contamination in RTE food products stored at 18 °C: 7.9% *E*. *coli*, 49.8% *B*. *cereus*, 17.9% *S*. *aureus*, and 42.7% *Pseudomonas* spp.^10^. Among the 4 major food types they tested (all stored at 18 °C), coliforms were found in 88% of ham samples, 80% of seafood, 72.7% of other meats, and 62.2% of vegetables. In a separate study, Wei *et al*. reported *S*. *aureus* contamination in 19% of commercial RTE food products purchased from traditional markets, 12.7% from supermarkets, and 9.5% from warehouse stores^12^. They also identified *E*. *coli* in 5–7% of all food samples obtained from supermarkets and warehouse stores. Neither one of these studies looked at antimicrobial susceptibility patterns or resistance mechanisms.

In the present study, the highest percentages of ceftazidime-resistant bacteria were isolated in RTE street food samples of cold noodles (43.3%), followed by fruit platters (17.8%) and spring rolls (16.7%) (Fig. 2B). Results from efforts to determine antimicrobial resistance patterns indicate unexpectedly high levels of multi-drug resistance (71.4–100%) (Fig. 3E). Low resistance was determined for levofloxacin (Fig. 3A–D), indicating that it should be considered for treating severe cases of food poisoning. While meropenem resistance levels were high in *Enterobacteriaceae* (13/15; 86.7%), *Acinetobacter* spp. (20/22; 90.9%), and *Pseudomonas* spp. (12/12; 100%) (Supplementary Table S2), carbapenemases were not detected in these isolates. Carbapenemases such as *bla*_KPC-2_ and *bla*_IMP-8_ tend to be detected in hospitalized patients, with low potential for widespread dissemination to communities or via RTE foods^29,30^.

Researchers studying different types of salads in Portugal and Switzerland have reported that RTE food pathogens share the same ESBL genes as clinical isolates collected from hospital patients, suggesting the potential for commensal bacteria to act as antibiotic-resistant gene reservoirs, especially in hospital settings^9,20^. Further, ESBL-producing *Enterobacteriaceae* isolates have been reported in beef burger, seafood, and raw vegetable samples^15,31,32^. Based on these reports, ESBL gene sources in human food chains include both RTE and non-RTE food products. In the present study we identified various ESBLs and plasmid-mediated *ampC* genes commonly found among bacteria isolates collected in Taiwanese hospitals^21–23^. These genes were especially abundant in ceftazidime non-susceptible non-fermenting gram-negative bacilli, mostly *Acinetobacter* spp. (Table 1). The data confirm the presence of antibiotic-resistant gene reservoirs in RTE street foods sold in Taiwan’s three largest cities, and that stepped-up surveillance efforts are required to monitor their movement.

Efflux pump activity has been associated with ceftazidime resistance^25,26^. Quantitative RT-PCR results indicate that the AdeIJK efflux pump contributed to the ceftazidime resistance that we observed in *Acinetobacter* spp. (Fig. 5). Specifically, among the 22 ceftazidime non-susceptible *Acinetobacter* spp. isolates we examined, 3 contained ESBL genes, 5 contained ESBL genes plus efflux pump activity, 11 efflux pump activity only, and 3 unknown resistance mechanisms. To our knowledge, this is the first report to demonstrate a link between the AdeIJK efflux pump and ceftazidime resistance in foodborne pathogens. One previous study reported that efflux pumps contributed to 94.4% of ceftazidime-resistant (17/18) and 72.7% of chloramphenicol-resistant (16/22) *Burkholderia cepacia* complex isolates^21^. Combined, these reports and data from the present study indicate that efflux pump activity plays an important role in antibiotic resistance mechanisms. The low ceftazidime resistance levels that we observed in *S*. *maltophilia* and *Enterobacteriaceae* isolates suggest that the ceftazidime resistance noted in this study is attributable to an unknown ESBL gene or different efflux pump type. We will attempt to confirm this finding and identify the gene and pump types in a future study.

Our results confirm the capacity of ready-to-eat street foods found in Taiwan, especially cold noodles, to act as antimicrobial-resistant bacteria reservoirs. Additional research efforts are required to quantify the antimicrobial-resistant bacteria that were isolated and analyzed in the present study. The potential for the horizontal transfer of resistance genes and the transmission of multidrug-resistant bacteria warrant further monitoring and control measures throughout Taiwan, but especially in densely populated urban areas. Unfortunately, one study concluded that special training in hygienic practices in Taiwan did not result in a sufficient increase in knowledge or effective practices among food vendors^33^. Clearly a renewed effort needs to be made to establish strict education standards for hygienic practices among street food vendors and food preparers, with local health officials monitoring such effort to ensure RTE food product safety.

## Methods

### Sample collection

A total of 270 food samples (90 spring rolls, 90 servings of cold noodles, and 90 fruit platters) were collected from randomly selected vendors operating in Kaohsiung, Taichung, and Taipei between June and November of 2014. All samples were transported to a single laboratory in their original packaging within 2 h (Kaohsiung and Taichung), or within 4 h at a slightly chilled temperature of 16 °C (Taipei).

### Microbiological analysis

Ten grams of each sample were homogenized in a stomacher blender, enriched using brain-heart infusion broth (BD, East Rutherford, NJ), and incubated overnight at 37 °C. Individual loopfuls of bacterial suspension were added to eosin-methylene blue (EMB) agar containing ceftazidime (8 μg/ml) and incubated overnight at 37 °C. When different colony morphologies were observed in the same food sample, all colonies were subcultured on EMB agar containing 8 μg/ml ceftazidime. Bacterial identification was performed using 16S rRNA gene sequencing as described in a previous study^30^. ESBL production was confirmed by tests recommended by the Clinical and Laboratory Standards Institute, using cefotaxime (30 mg) and ceftazidime (30 mg) disks alone and in combination with clavulanic acid (10 mg) (Becton, Dickinson, MD)^34^. Positive phenotypes were defined as growth-inhibitory zones from either cefotaxime or ceftazidime disks plus clavulanic acid ≥5 mm of growth-inhibitory zones from either cefotaxime or ceftazidime disks.

### Antimicrobial susceptibility

These tests were performed using agar dilution methods according to Clinical and Laboratory Standards Institute guidelines^34^. Minimum inhibitory concentration (MIC) was defined as the lowest concentration of an antibiotic preventing bacterial growth after 16 to 20 h of incubation at 37 °C. We tested 11 antimicrobial agents: cefotaxime, ceftazidime, chloramphenicol, colistin, gentamicin, levofloxacin, meropenem, ticarcillin, ticarcillin-clavulanic acid, tetracycline, and trimethoprim-sulphamethoxazole.

### Pulsed-field gel electrophoresis (PFGE)

PFGE typing of restriction enzyme-digested DNA (New England BioLabs, Ipswich, MA) was prepared as previously described^21^. *Enterobacteriaceae* and *S*. *maltophilia* were digested with XbaI; *Pseudomonas* spp. and *Acinetobacter* spp. were digested with SpeI and ApaI, respectively. Restriction fragments ranging from 50 to 500 kb were separated using a CHEF Mapper apparatus (Bio-Rad) for 20 h at 200 V and 14 °C. Gels were stained with ethidium bromide and photographed under UV light. Dice similarity indices were used to construct pulsotype relationship dendrogrammes by an unweighted pair group method using arithmetic averages (UPGMA) with BioNumerics software (v.6.5, Applied Maths). Based on dendrogramme results, pulsotypes were assigned to the same cluster if they exhibited at least 80% similarity.

### Detection of antibiotic resistance genes

Plasmid DNA was extracted using a QIAGEN Plasmid Mini Kit. ESBLs (*bla*_SHV_, *bla*_TEM_ *, bla*_CTX-M-G1_, *bla*_CTX-M-G2_, *bla*_CTX-M-G9_, *bla*_PER_, *bla*_VEB_); OXA genes (*bla*_OXA-1_, *bla*_OXA-2_, *bla*_OXA-9_, and *bla*_OXA-10_ genes); plasmid-mediated AmpC genes (*bla*_DHA_ and *bla*_CMY_)^23,30,35,36^; carbapenemases (*bla*_KPC_, *bla*_NDM_, *bla*_VIM_, *bla*_IMP_, *bla*_NMC_, *bla*_SME_, *bla*_SPM-1_, *bla*_GIM-1_, *bla*_SIM-1_, *bla*_IMI_, *bla*_GES_, and *bla*_OXA-48_)^30^; chloramphenicol resistance genes (*catI*, *catII*, *catIII* and *cmlA*)^37^; tetracycline resistance genes (*tet*(A)*, tet*(B), *tet*(C)*, tet*(D)*, tet*(E) and *tet*(G)); and gentamicin resistance genes (*aacC1, aacC2*, *aacC3*, and *aacC4*)^38^ were detected by PCR, using primer sets listed in previous reports (Supplementary Table S1). Positive controls for these resistance genes were included in all PCR analyses.

### Efflux pump activity assays

To evaluate the contributions of efflux pump activity to ceftazidime resistance among the isolates, MIC resistance patterns were determined via Mueller-Hinton agar dilution with and without 12.5 μM of the efflux pump inhibitor carbonyl cyanide-m-chlorophenyl hydrazone (CCCP; Sigma- Aldrich, St. Louis, MO)^39^. Bacterial isolates containing ceftazidime resistance-associated efflux pumps were identified as any CCCP-inoculated strain exhibiting a minimum 4-fold decrease in MIC.

### Quantitative RNA expression for efflux pumps

RNA extraction and cDNA synthesis were performed as described in our previous study^21^. PCR reactions took place in buffer containing 1X FastStart Universal SYBR Green Master (Roche), 300 nM primers, and 2 μl cDNA in an ABI7000 machine following manufacturer instructions (96-well plates). AdeIJK and AdeDE efflux pumps have previously been described as contributing to ceftazidime resistance in *Acinetobacter* spp.^26,40^. Quantitative RNA expression of *adeI* and *adeE* was analyzed using the primers listed in Supplementary Table S1 ^40^. Relative fold changes in the transcript levels of indicated genes were normalized to the 16S rDNA gene (internal control) and calculated using the 2^*−ΔΔCT*^ method. Non-parametric Mann-Whitney U tests were used to examine differences in quantitative RNA expressions of *adeI* and *adeE* between test (ceftazidime resistance-associated efflux pumps) and control strains (no efflux pump activity). Statistical significance was established as *p* < 0.05.

## Electronic supplementary material

SUPPLEMENTARY table and figure
