# Supplementary material for: Antimicrobial resistance and genetic diversity in ceftazidime non-susceptible bacterial pathogens from ready-to-eat street foods in three Taiwanese cities
Source: Sci Rep. 2017 Nov 14;7:15515. doi: 10.1038/s41598-017-15627-8 (PMC5686198; doi:10.1038/s41598-017-15627-8)

Antimicrobial Resistance and Genetic Diversity of Ceftazidime Non-susceptible Bacterial Pathogens from Ready-to-eat Street Foods in Three Taiwanese Cities

Lin Lin [1], Sheng-Fan Wang [2], Tsung-Ying Yang [2], Wei-Chun Hung [3], Min-Yu Chan [2], Sung-Pin Tseng [2,4*]

[1] Department of Culinary Art, I-Shou University, Kaohsiung, Taiwan

[2] Department of Medical Laboratory Science and Biotechnology, Kaohsiung Medical University, Kaohsiung, Taiwan

[3] Department of Microbiology and Immunology, Kaohsiung Medical University, Kaohsiung, Taiwan

[4] Department of Marine Biotechnology and Resources, National Sun Yat-sen University, Kaohsiung, Taiwan

[*]**Corresponding author:** Sung-Pin Tseng

100, Shih-Chuan 1st Road, Kaohsiung, Taiwan

Department of Medical Laboratory Science and Biotechnology, Kaohsiung Medical University

Phone: (886) 7-3121101 ext. 2353, Fax: (886) 7-3113449

Email: tsengsp@kmu.edu.tw

Table S1. Primers used in this study.

Target Gene:	Primer Sequence (5’→3’)		Annealing Temp (ºC)	Amplicon Size (bp)	Ref
	Forward	Reverse			
**ESBL genes**					
*bla*SHV	GGGTTATTCTTATTTGTCGC	TTAGCGTTGCCAGTGCTC	52	927	30
*bla*TEM	GACAGTTACCAATGCTTAATCA	ATAAAATTCTTGAAGACGAAA	55	1080	30
*bla*CTX-M-G1	GGTTAAAAAATCACTGCGTC	TTGGTGACGATTTTAGCCGC	55	864	30
*bla*CTX-M-G2	ATGATGACTCAGAGCATTCG	TGGGTTACGATTTTCGCCGC	55	866	30
*bla*CTX-M-G9	ATGGTGACAAAGAGAGTGCA	CCCTTCGGCGATGATTCTC	55	870	30
*bla*PER	GCAACTGCTGCAATACTCGG	ATGTGCGACCACAGTACCAG	50	338	35
*bla*VEB	CGACTTCCATTTCCCGATGC	GGACTCTGCAACAAATACGC	50	641	35
*bla*OXA-1	ACACAATACATATCAACTTCGC	AGTGTGTTTAGAATGGTGATC	45.6	812	36
*bla*OXA-2	TTCAAGCCAAAGGCACGATAG	TCCGAGTTGACTGCCGGGTTG	52	701	36
*bla*OXA-9	CGTCGCTCACCATATCTCCC	CCTCTCGTGCTTTAGACCCG	52	313	36
*bla*OXA-10	CGTGCTTTGTAAAAGTAGCAG	CATGATTTTGGTGGGAATGG	46	652	23
**Plasmid-mediated AmpC genes**					
*bla*DHA	CTGATGAAAAAATCGTTATC	ATTCCAGTGCACTCAAAATA	55	1136	30
*bla*CMY	CTGCTGCTGACAGCCTCTTT	TTTTCAAGAATGCGCCAGGC	55	1109	30
**Carbapenemase genes**					
*bla*KPC	ATGTCACTGTATCGCCGTCT	TTTTCAGAGCCTTACTGCCC	52	893	30
*bla*NDM	GGTTTGGCGATCTGGTTTTC	CGGAATGGCTCATCACGATC	54	1133	30
*bla*VIM-1	TTATGGAGCAGCAACCGATGT	CAAAAGTCCCGCTCCAACGA	52	920	30
*bla*VIM-2	AAAGTTATGCCGCACTCACC	TGCAACTTCATGTTATGCCG	50	865	30
*bla*IMP-1	TGAGCAAGTTATCTGTATTC	TTAGTTGCTTGGTTTTGATG	50	738	30
*bla*IMP-2	GGCAGTCGCCCTAAAACAAA	TAGTTACTTGGCTGTGATGG	50	737	30
*bla*NMC	GCATTGATATACCTTTAGCAGAGA	CGGTGATAAAATCACACTGAGCATA	52	2158	30
*bla*SME	AGATAGTAAATTTTATAG	CTCTAACGCTAATAG	42	1138	30
*bla*SPM-1	CCTACAATCTAACGGCGACC	TCGCCGTGTCCAGGTATAAC	54	650	30
*bla*GIM-1	AGAACCTTGACCGAACGCAG	ACTCATGACTCCTCACGAGG	54	748	30
*bla*SIM-1	TACAAGGGATTCGGCATCG	TAATGGCCTGTTCCCATGTG	51	569	30

*bla*IMI	ATAGCCATCCTTGTTTAGCTC	TCTGCGATTACTTTATCCTC	50	818	30
*bla*GES	GTTTTGCAATGTGCTCAACG	TGCCATAGCAATAGGCGTAG	52	371	30
*bla*OXA-48	CAAAGGAATGGCAAGAAAACAAAA	GCGCAGCCCTAAACCATCC	55	798	30
**Chloramphenicol-resistant genes**					
catI	AGTTGCTCAATGTACCTATAACC	TTGTAATTCATTAAGCATTCTGCC	46.1	585	37
catII	ACACTTTGCCCTTTATCGTC	TGAAAGCCATCACATACTGC	44.7	495	37
catIII	TTCGCCGTGAGCATTTTG	TCGGATGAGTATGGGCAAC	44.5	508	37
cmlA	CCGCCACGGTGTTGTTGTTATC	CACCTTGCCTGCCCATCATTAG	51.7	698	37
**Tetracycline-resistant genes**					
tet(A)	GCTACATCCTGCTTGCCTTC	CATAGATCGCCGTGAAGAGG	48.8	210	37
tet(B)	TTGGTTAGGGGCAAGTTTTG	GTAATGGGCCAATAACACCG	45.7	659	37
tet(C)	CTTGAGAGCCTTCAACCCAG	ATGGTCCTCATCTACCTGCC	48.8	418	37
tet(D)	AAACCATTACGGCATTCTGC	GACCGGATACACCATCCATC	46.7	787	37
tet(E)	AAACCACATCCTCCATACGC	AAATAGGCCACAACCGTCAG	46.8	278	37
tet(G)	GCTCGGTGGTATCTCTGCTC	AGCAACAGAATCGGGAACAC	48.8	210	37
**Gentamicin-resistant genes**					
aac(3)-I	ACCTACTCCCAACATCAGCC	ATATAGATCTCACTACGCGC	46.7	169	38
aac(3)-II	ACTGTGATGGGATACGCGTC	CTCCGTCAGCGTTTCAGCTA	48.8	237	38
aac(3)-III	CACAAGAACGTGGTCCGCTA	AACAGGTAAGCATCCGCATC	47.8	185	38
aac(3)-IV	CTTCAGGATGGCAAGTTGGT	TCATCTCGTTCTCCGCTCAT	46.8	286	38
**qRT-PCR**					
16SrDNA	TCCTCCAGTTTGTCACTGGC	GTCAGCTCGTGTCGTGAGAT	62	116	This study
adeE [a]	GTAGTAGTTCGGCAGGACAA	GCGGTTCTAACATCTGATGG	60	376	40
adeI [b]	CAAGTTGCAGCAGCTAAGGC	GCAGTAACCAAAGCACCAGC	62	125	This study

[a] used for detecting AdeDE efflux pump.

[b] used for detecting AdeIJK efflux pump.

Table S2. Antimicrobial susceptibilities of 70 bacterial isolates.

Antibiotic \ Species	*Enterobacteriaceae* (n=15) Number (%) of isolates			*Acinetobacter* spp. (n=22) Number (%) of isolates			*Pseudomonas* spp. (n=12) Number (%) of isolates			*Stenotrophomonas maltophilia* (n=21) Number (%) of isolates		
	S	I	R	S	I	R	S	I	R	S	I	R
Cefotaxime	0 (0)	0 (0)	15 (100)	0 (0)	2 (9.1)	20 (90.9)	0 (0)	0 (0)	12 (100)	ND	ND	ND
Ceftazidime	0 (0)	4 (26.7)	11 (73.3)	0 (0)	6 (27.3)	16 (72.7)	0 (0)	2 (16.7)	10 (83.3)	0 (0)	2 (9.5)	19 (90.5)
Chloramphenicol	1 (6.7)	0 (0)	14 (93.3)	ND	ND	ND	0 (0)	0 (0)	12 (100)	0 (0)	0 (0)	21 (100)
Colistin	ND[d]	ND	ND	2 (9.1)	0 (0)	20 (90.9)	1 (8.3)	0 (0)	11 (91.7)	ND	ND	ND
Gentamicin	3 (20.0)	0 (0)	12 (80.0)	3 (13.6)	0 (0)	19 (86.4)	2 (16.7)	1 (8.3)	9 (75.0)	ND	ND	ND
Levofloxacin	9 (60.0)	3 (20.0)	3 (20.0)	15 (68.2)	1 (4.5)	6 (27.3)	12 (100)	0 (0)	0 (0)	18 (85.7)	2 (9.5)	1 (4.8)
Meropenem	2 (13.3)	0 (0)	13 (86.7)	1 (4.5)	1(4.5)	20 (90.9)	0 (0)	0 (0)	12 (100)	ND	ND	ND
Streptomycin	ND	ND	ND	ND	ND	ND	ND	ND	ND	ND	ND	ND
Ticarcillin	0 (0)	2 (13.3)	13 (86.7)	0 (0)	7 (31.8)	15 (68.2)	0 (0)	2 (16.7)	10 (83.3)	ND	ND	ND
Ticarcillin-clavulanic acid	0 (0)	5 (33.3)	10 (66.7)	5 (22.7)	8 (36.4)	9 (40.9)	0 (0)	3 (25.0)	9 (75.0)	7 (33.3)	0 (0)	14 (66.7)
Tetracycline	2 (13.3)	2 (13.3)	11 (73.4)	2 (9.1)	2 (9.1)	18 (81.8)	0 (0)	1 (8.3)	11 (91.7)	ND	ND	ND
Trimethoprim-sulfamethoxazole	7 (46.7)	0 (0)	8 (53.3)	9 (40.9)	0 (0)	13 (59.1)	0 (0)	0 (0)	12 (100)	6 (28.6)	0 (0)	15 (71.4)

S, susceptible.

I, intermediate.

R, resistant.

ND, criteria for antibiotic resistance not defined by the Clinical Laboratory Standards Institute (CLSI).

Supplementary Fig S1. ***Acinetobacter*** **spp. isolates (n=22). Dendrogram of pulsotype relationships identified by unweighted pair group method with arithmetic mean (UPGMA) (BioNumerics v6.5, Applied Maths). Pulsotypes exhibiting 80% similarity were assigned to the same cluster. KA: Kaohsiung, TC: Taichung, TP: Taipei.**

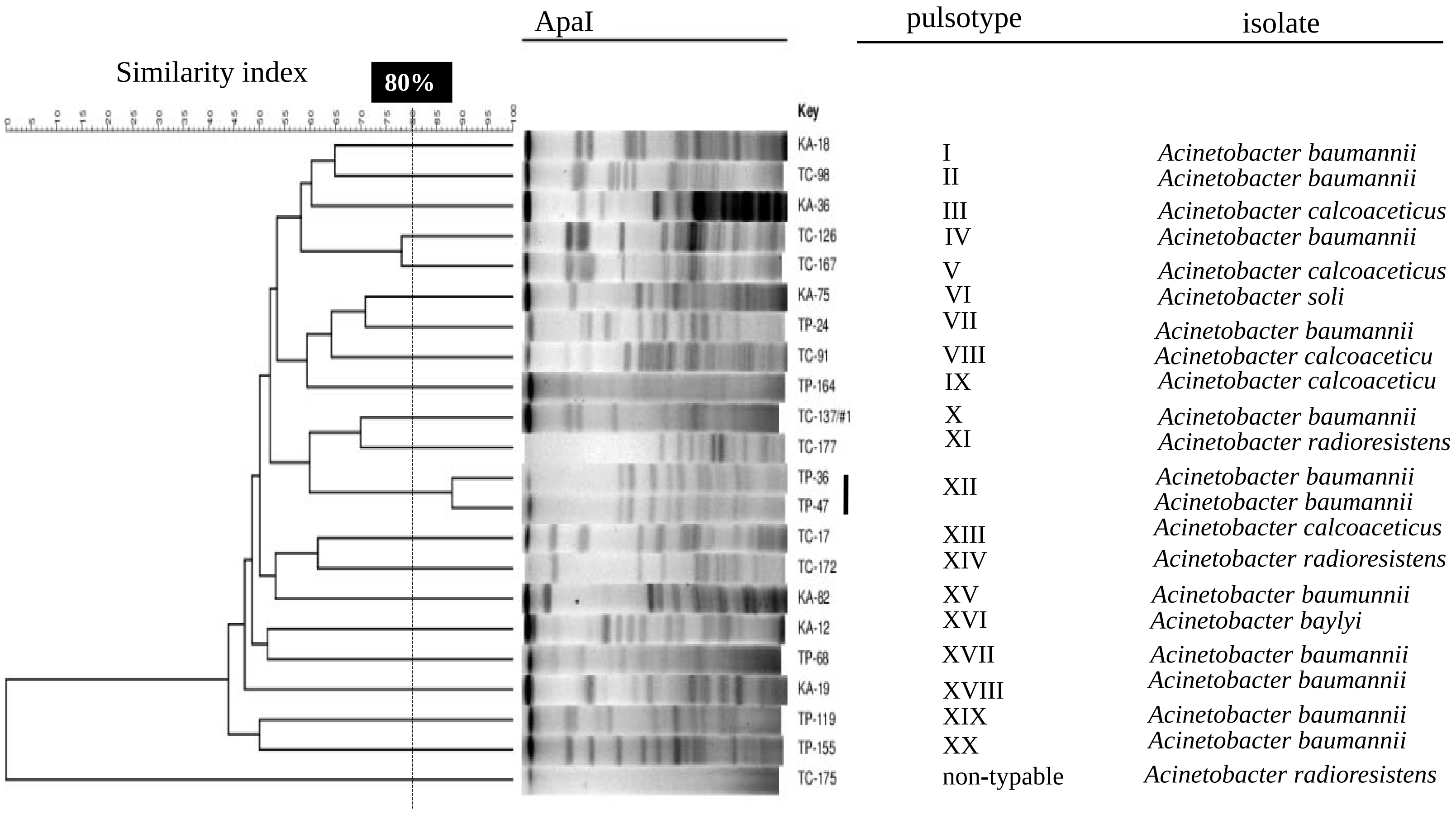

Supplementary Fig S2. *Pseudomonas* spp. isolates (n=12). Dendrogram of pulsotype relationships identified by unweighted pair group method with arithmetic mean (UPGMA) (BioNumerics v6.5, Applied Maths). Pulsotypes exhibiting 80% similarity were assigned to the same cluster. KA: Kaohsiung, TC: Taichung, TP: Taipei.

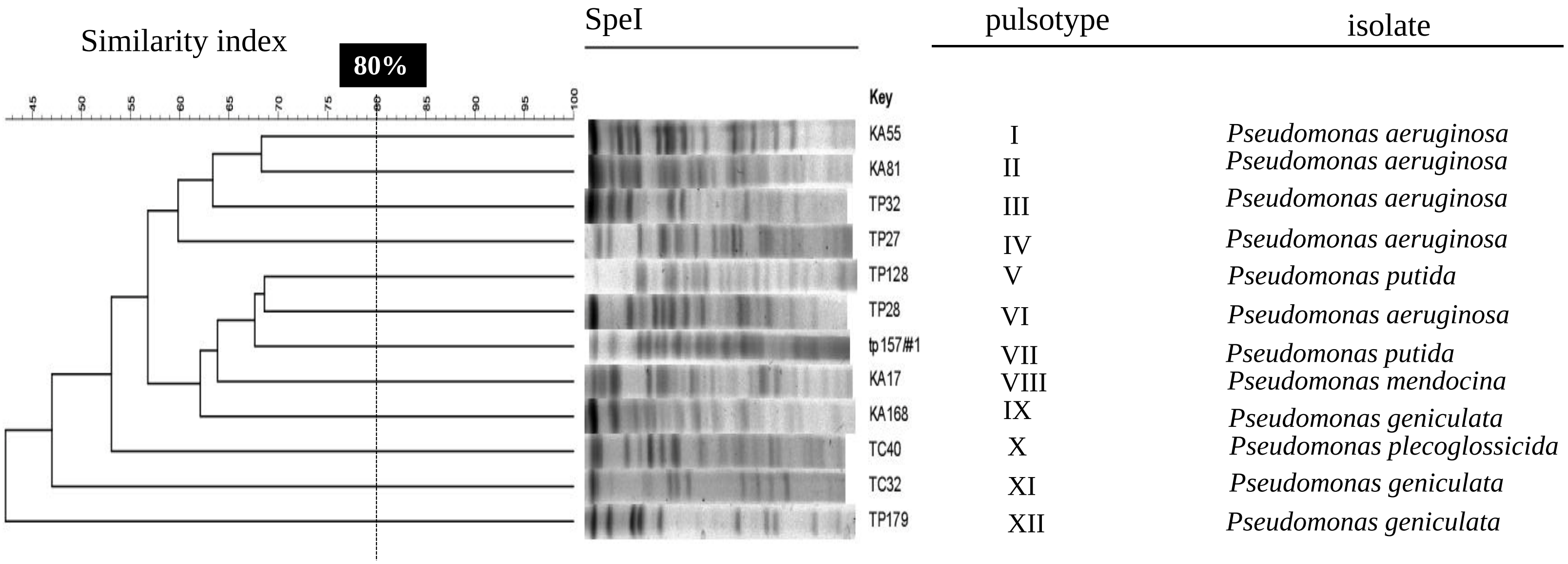

Supplementary Fig S3. *S. maltophilia* isolates (n=21). Dendrogram of pulsotype relationships identified by unweighted pair group method with arithmetic mean (UPGMA) (BioNumerics v6.5, Applied Maths). Pulsotypes exhibiting 80% similarity were assigned to the same cluster. KA: Kaohsiung, TC: Taichung, TP: Taipei.

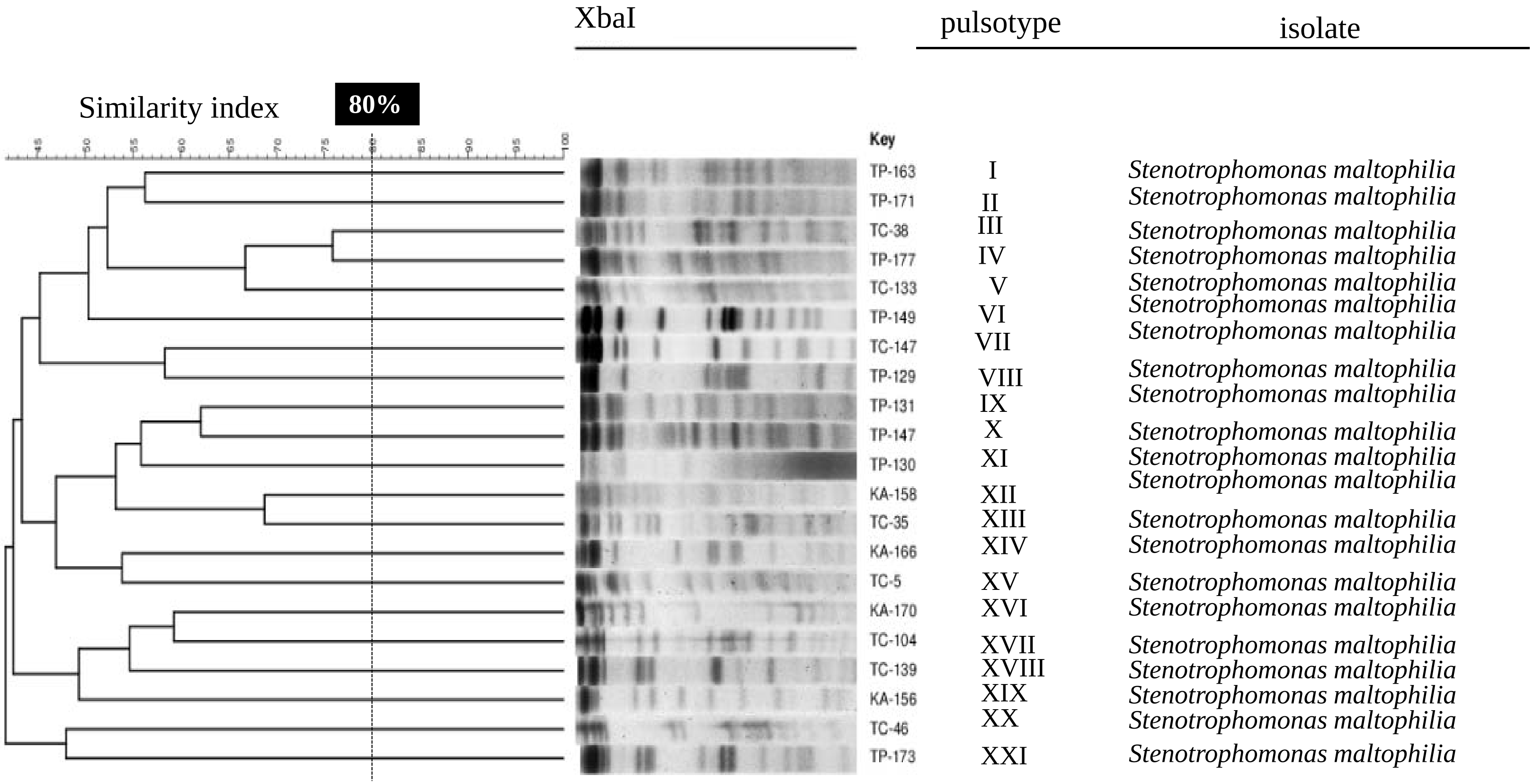

Supplementary Fig S4. *Enterobacteriaceae* isolates (n=15). Dendrogram of pulsotype relationships identified by unweighted pair group method with arithmetic mean (UPGMA) (BioNumerics v6.5, Applied Maths). Pulsotypes exhibiting 80% similarity were assigned to the same cluster. KA: Kaohsiung, TC: Taichung, TP: Taipei.

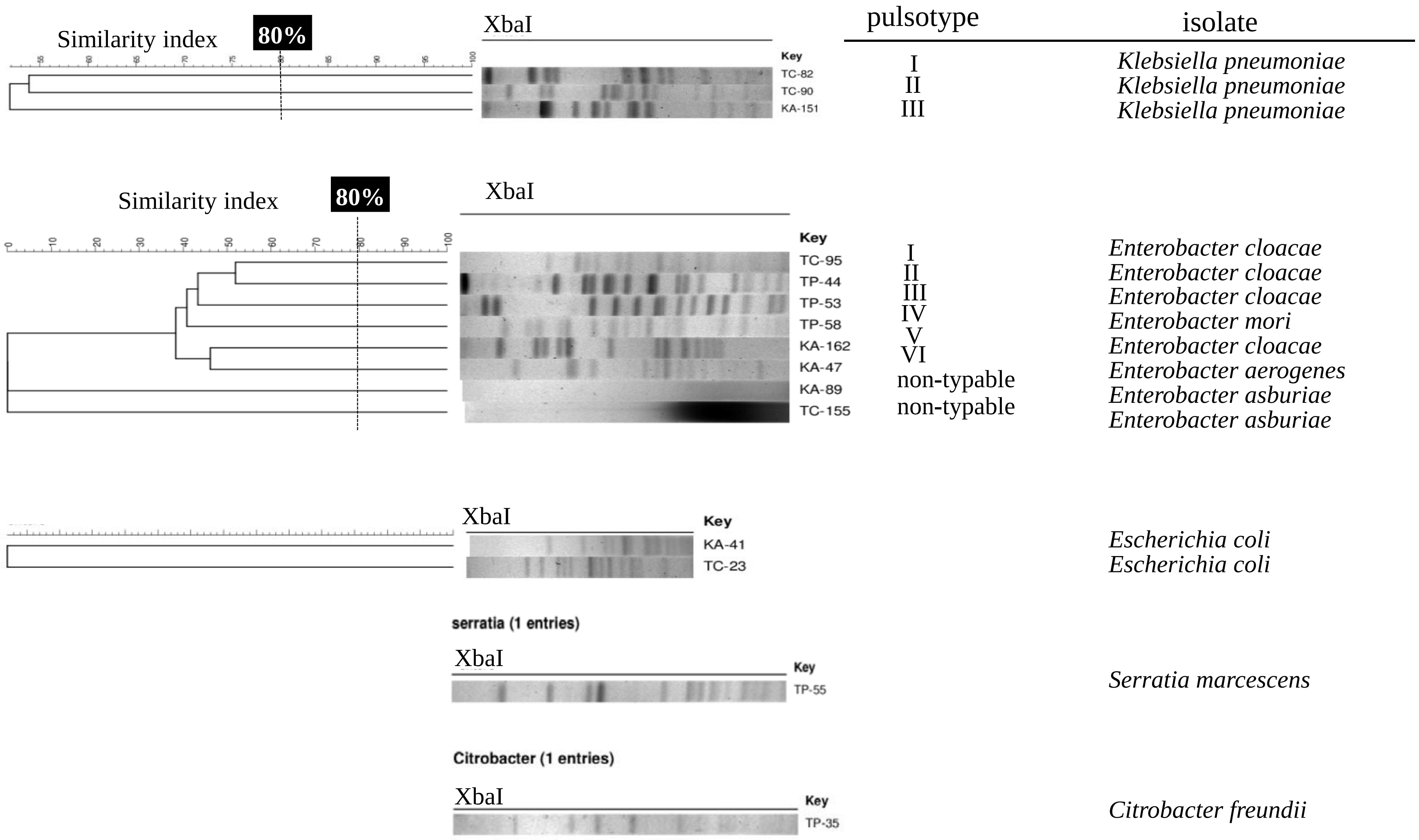

Supplement: Supplementary file 1 — SUPPLEMENTARY table and figure [file 41598_2017_15627_MOESM1_ESM.pdf]
